# Chemoattractant-Induced Intracellular Calcium Responses Using a Calcium Biosensor GCaMP3 in *Dictyostelium discoideum*

**DOI:** 10.4014/jmb.2607.07011

**Published:** 2026-10-21

**Authors:** Dahyeon Kim, Hun Jeong, Uri Han, Nara Han, Taeck Joong Jeon

**Affiliations:** 1 Department of Integrative Biological Sciences & BK21 FOUR Educational Research Group for Age-Associated Disorder Control Technology, Chosun University, Gwangju, Republic of Korea; 2 The Basic Science Institute of Chosun University, Chosun University, Gwangju, Republic of Korea

**Keywords:** Calcium signaling, GCaMP, chemotaxis, *Dictyostelium*, IplA, cAMP

## Abstract

Calcium (Ca^2+^) is a critical second messenger in eukaryotic chemotaxis and development, yet the molecular coordination of its spatiotemporal dynamics across different chemoattractant systems remains incompletely understood. Using the genetically encoded calcium indicator GCaMP3, we characterized real-time intracellular calcium ([Ca^2+^]i) dynamics in *Dictyostelium discoideum* under two developmental stages. Both cAMP-stimulated developed cells and folate-stimulated vegetative cells exhibited a rapid, transient [Ca^2+^]i elevation peaking at approximately 12 sec, strictly dependent on extracellular Ca^2+^ influx. Genetic and pharmacological analyses using signaling mutants (*Gβ-*, *GCA/sGC-*, *IplA-*, *PlaA*-null) revealed a universal requirement for IplA and for SERCA-dependent ER calcium sequestration, consistent with a store-operated calcium entry (SOCE)-like mechanism. Strikingly, folate-stimulated vegetative *Gβ-*null cells displayed an accelerated [Ca^2+^]i transient peaking at 8-12 sec, suggesting a G-protein-independent Ca^2+^ entry mechanism. cGMP and calmodulin act as downstream kinetic modulators rather than upstream gating factors. Together, these findings advance our understanding of the molecular architecture of chemoattractant-induced calcium signaling in *Dictyostelium*.

## Introduction

Calcium (Ca^2+^) is a ubiquitous intracellular second messenger that governs diverse cellular processes, including cell proliferation, apoptosis, secretion, and directed cell migration [1, 2]. In non-excitable eukaryotic cells, cytosolic Ca^2+^ concentration ([Ca^2+^]i) is maintained at a resting level of approximately 100 nM and transiently elevated in response to extracellular stimuli through two complementary mechanisms: (1) release from intracellular stores—principally the endoplasmic reticulum (ER)—through inositol 1,4,5-trisphosphate receptors (IP_3_Rs) or ryanodine receptors (RyRs); and (2) plasma membrane Ca^2+^ influx via store-operated Ca^2+^ entry (SOCE) channels, driven by ER store depletion through the STIM1/Orai1 complex [1-3]. These precisely regulated Ca^2+^ transients couple extracellular stimuli to intracellular effectors and are critical for chemotaxis, cell migration, and developmental signaling [1-3].

The social amoeba *Dictyostelium discoideum* is a genetically tractable model system for studying Ca^2+^ signaling in the context of chemotaxis and multicellular development [4, 5]. During starvation-induced development, *Dictyostelium* cells aggregate in response to periodic cAMP waves detected by surface receptors (cAR1–cAR4) coupled to heterotrimeric G-proteins. In vegetative cells, folate acts as the primary chemoattractant through distinct receptor systems. Cytosolic [Ca^2+^]i oscillates periodically in synchrony with cAMP relay waves during aggregation, demonstrating tight integration between Ca^2+^ and chemoattractant signaling [4]. Both chemoattractant systems have been reported to elevate [Ca^2+^]i transiently, although the precise molecular mechanisms and whether they share common downstream effectors have remained incompletely defined.

Historically, Ca^2+^ dynamics in *Dictyostelium* were measured using radioisotope flux assays or chemical fluorescent dyes such as Fura-2, which provided bulk population-level measurements with limited spatial and temporal resolution [4]. The development of genetically encoded Ca^2+^ indicators (GECIs), particularly the GCaMP series, has transformed live-cell Ca^2+^ imaging by providing high sensitivity, fast kinetics, and compatibility with standard fluorescence microscopy. GCaMP3, developed by Tian *et al*. (2009), offered substantially improved signal-to-noise ratio over earlier GECIs, enabling stable expression and real-time imaging in diverse model organisms [6]. A recent study by Kim *et al*. (2025) established pDXA-GCaMP3 as a *Dictyostelium*-specific expression system and demonstrated that GCaMP3 can faithfully report [Ca^2+^]i dynamics in response to external Ca^2+^ stimuli in this organism, with distinct responses in vegetative versus aggregation-competent cells [7].

Several key molecular components of Ca^2+^ signaling in *Dictyostelium* have been identified through genetic studies [4, 8, 9]. The Gβ subunit of the heterotrimeric G-protein complex is a central transducer of cAMP receptor signaling, and its deletion abolishes the majority of downstream chemotactic responses. The IplA protein, a distant homolog of mammalian IP_3_Rs, has been implicated in intracellular Ca^2+^ release and is required for thermotaxis and electrotaxis; however, its precise role in chemoattractant-induced Ca^2+^ responses remains poorly characterized [4]. Furthermore, the dual-function guanylyl cyclases GCA and sGC produce cGMP, a second messenger that regulates myosin II-dependent motility, and may indirectly influence Ca^2+^ channel activity [10, 11]. The role of phospholipase A (PlaA) in Ca^2+^ mobilization has also been suggested but not rigorously tested [12]. Importantly, the relationship between ER Ca^2+^ stores and plasma membrane Ca^2+^ influx—specifically whether SOCE-like mechanisms operate in *Dictyostelium*—has not been directly demonstrated, despite indirect evidence from pharmacological studies using SERCA inhibitors such as thapsigargin and BHQ [13].

In this study, we employed GCaMP3 to characterize in real time the chemoattractant-induced [Ca^2+^]i dynamics in *Dictyostelium*. Using a panel of genetic mutants—including cells deficient in Gβ, GCA/sGC, IplA, and PlaA—combined with defined pharmacological perturbations (EGTA, BHQ, W7), we systematically dissected the contributions of extracellular Ca^2+^ influx, ER Ca^2+^ stores, G-protein signaling, and calmodulin activity to cAMP- and folate-induced Ca^2+^ responses across two distinct developmental stages. Our results show that IplA and SERCA-dependent ER calcium sequestration is essential in both chemoattractant systems, and also suggest the existence of a G-protein-independent Ca^2+^ influx mechanism in vegetative cells.

## Materials and Methods

### Cell Strains and Culture Conditions

*Dictyostelium* wild-type strain KAx3 (DBS0236487), *Gβ-*null cells (DBS0236531, lacking the G-protein β-subunit, *gpbA*^−^), *gca/sgc*-null cells (DBS0236000, lacking both soluble and membrane-bound guanylyl cyclases), *iplA*-null cells (DBS0266326, lacking the IP_3_R-like channel IplA), and *plaA*-null cells (DBS0238068, lacking phospholipase A2) were obtained from the dictyBase stock center (USA). *Dictyostelium* cells were cultured axenically at 22°C in HL5 media or in association with *Klebsiella aerogenes* at 22°C. The knock-out strains and transformants were maintained in 10 μg/mL blasticidin or G418 (Geneticin; Sigma-Aldrich, USA).

The GCaMP3 coding sequence was subcloned into the *Dictyostelium* expression vector pDXA-3H to generate pDXA-GCaMP3, as previously described [7]. Plasmids were introduced into wild-type and mutant *Dictyostelium* cells by electroporation, followed by the addition of G418 to 20 μg/mL for selection of stable transformants. Expression of GCaMP3 was confirmed by immunofluorescence and immunoblotting with anti-GFP antibodies.

The following pharmacological agents were used to dissect the contributions of specific Ca^2+^ handling mechanisms: 2,5-Di-tert-butylhydroquinone (BHQ), a SERCA inhibitor, and W7 (N-(6-aminohexyl)-5-chloro-1-naphthalenesulfonamide), a calmodulin antagonist, were purchased from MedChemExpress (USA). Dimethyl sulfoxide (DMSO) was purchased from Sigma-Aldrich. Stock solutions of BHQ (200 mM) and W7 (30 mM) were prepared in DMSO, respectively. Cells were incubated with BHQ or W7 for 1 h at 22°C prior to imaging at a final concentration of 50 μM. EGTA (ethylene glycol-bis-tetra acetic acid), a membrane-impermeant Ca^2+^ chelator to eliminate extracellular free Ca^2+^ was added directly to DB buffer at a final concentration of 5 mM.

### Preparation of Aggregation-Competent (developed) Cells and Vegetative Cells

For experiments using cAMP as the chemoattractant, *Dictyostelium* cells were developed to aggregation competency by starvation in phosphate buffer. Vegetative cells at a density of 2–5 × 10^6^ cells/mL were harvested by centrifugation at 500 × g for 3 min at room temperature, washed twice with DB buffer (5 mM Na_2_HPO_4_, 5 mM KH_2_PO_4_, 2 mM MgCl_2_, and 1 mM CaCl_2_, pH 6.5), and resuspended in DB buffer at 5 × 10^6^ cells/mL. Cells were then pulsed with 30 nM cAMP at 6-minute intervals for 5-6 h at 22°C with gentle rotation (150 rpm) to induce expression of cAMP receptors and aggregation-associated signaling machinery. Aggregation-competent cells prepared in this manner were used immediately for calcium imaging experiments involving cAMP stimulation.

For experiments using folate as the chemoattractant, vegetative cells maintained in HL5 medium were used directly without starvation, as folate chemotaxis occurs in the vegetative growth phase. Cells were harvested, washed twice with DB buffer to remove HL5 medium components, and resuspended in DB buffer at 1 × 10^6^ cells/mL for imaging.

### Uniform Chemoattractant Stimulation and Image Acquisition

Cyclic AMP (cAMP; Sigma-Aldrich) stock solution (150 μM) was prepared by dissolving cAMP in DB buffer. For uniform stimulation, the stock solution (1/10 volume) was rapidly and evenly pipetted into the imaging dish to achieve a final concentration of 15 μM cAMP [14]. Folate (folic acid; Sigma-Aldrich) stock solution (1 mM) was prepared in Na/K buffer or DB buffer, and added to the imaging dish to a final concentration of 100 μM for stimulation of vegetative cells.

For each imaging experiment, developed or vegetative cells expressing GCaMP3 or empty GFP were harvested and resuspended in the appropriate buffer at 5 × 10^6^ cells/mL. A 100–200 μL aliquot of the cell suspension was seeded onto a 35 mm glass-bottom imaging dish and allowed to adhere to the substratum for 10–15 min at 22°C. For pharmacological experiments, BHQ or W7 was added to the adherent cells in the appropriate buffer and incubated for 1 h at 22°C prior to imaging.

All fluorescence images were acquired using an inverted fluorescence microscope (IX71; Olympus, Japan) equipped with a DS-Fi1 charge-coupled device (CCD) camera (Nikon, Japan) using NIS-Elements software (Nikon). For time-lapse recording, fluorescence images were acquired at 2-sec intervals for 1 min. Representative images at the indicated time points (0, 10, 20, and 40 sec) are presented in figures.

### Image Analysis

Fluorescence intensities were measured and quantified from time-lapse recordings using the Region of Interest (ROI) manager tool in ImageJ software (NIH, USA). For each cell, an ROI was manually drawn, and mean fluorescence intensity was measured for each time point throughout the recording. Background fluorescence was measured from cell-free regions of the same frame and subtracted from the cell ROI measurements. Relative fluorescence intensities were calculated by dividing the intensity at each time point (Et) by the intensity before stimulation (Eo) [7,14].

### Statistical Analysis

For each figure panel, the number of independent biological replicates and the total number of individual cells analyzed (N) are reported in the corresponding figure legend below. Peak amplitude (maximal Et/Eo) and time-to-peak were extracted for each cell and averaged first within each independent experiment (experiment-level means), and these experiment-level means (n = 3 independent experiments per condition) were used for statistical comparison, avoiding pseudoreplication at the single-cell level. For time-course comparisons among three or more conditions/genotypes (Figs. 1B, 1D, 2B, and 4B), one-way ANOVA with Tukey's post hoc test was additionally performed independently at each 2-second time point using the experiment-level mean fluorescence intensities, and the time windows over which statistically significant differences (*P* < 0.05) were observed are reported in the corresponding figure legends. For pairwise comparisons of time-course data (Fig. 3, Supplementary Fig. S1), two-tailed Student's t-test was applied at each time point using experiment-level means, and the results are reported in the corresponding figure legends.

## Results

### cAMP Stimulation Induces a Transient [Ca^2+^]i Elevation in Developed Wild-Type Cells That Is Strictly Dependent on Extracellular Ca^2+^

To monitor chemoattractant-induced Ca^2+^ dynamics in real time, we expressed GCaMP3 in developed (aggregation-competent) *Dictyostelium* cells using the pDXA expression system [7] and recorded fluorescence changes following uniform stimulation with 15 μM cAMP. In standard DB buffer containing 1 mM CaCl_2_ and 2 mM MgCl_2_, wild-type cells exhibited a rapid and transient increase in GCaMP fluorescence, with peak [Ca^2+^]i observed at approximately 12 sec post-stimulation, after which fluorescence returned toward baseline within 50 sec (Fig. 1A and 1B). No fluorescence change was observed in empty GFP-expressing control cells or in buffer-only negative controls, validating the specificity of the GCaMP signal. These response kinetics are consistent with previous aequorin-based measurements in *Dictyostelium* [10, 13] and demonstrate that GCaMP3 faithfully resolves the rapid [Ca^2+^]i transient at the single-cell level [7]. One-way ANOVA performed at each 2-sec time point confirmed that the GCaMP3+cAMP condition differed significantly from the GFP and buffer controls at 12–14 sec post-stimulation (*P* < 0.05). To determine the source of the Ca^2+^ signal, we stimulated cells under conditions that eliminate or chelate extracellular Ca^2+^ (Fig. 1C and 1D). Cells stimulated in Na/K phosphate buffer (lacking Ca^2+^), in DB buffer supplemented with 5 mM EGTA, or in Ca^2+^-depleted DB buffer all showed a complete absence of GCaMP fluorescence response to cAMP. These results indicate that extracellular Ca^2+^ influx across the plasma membrane is the obligate source of cAMP-evoked [Ca^2+^]i elevation. Cells stimulated in DB buffer lacking MgCl_2_ showed a numerically higher Ca^2+^ response compared to standard conditions (Fig. 1C and 1D). One-way ANOVA at each 2-sec time point indicated statistically significant differences among these buffer conditions at 10–14 sec post-stimulation (*P* < 0.05); however, the enhanced response in Mg^2+^-free buffer was not significantly different from that in standard DB buffer at any individual time point (*P* > 0.05).

### Gβ and IplA Are Essential for cAMP-Induced Ca^2+^ Responses, Whereas cGMP Signaling Modulates Response Kinetics

To identify the molecular components mediating cAMP-evoked Ca^2+^ responses in developed cells, we expressed GCaMP3 in cells deficient in Gβ (G-protein β-subunit), GCA/sGC (dual guanylyl cyclases), IplA (IP_3_R homolog), and PlaA (phospholipase A) and monitored [Ca^2+^]i dynamics in response to 15 μM cAMP (Fig. 2A and 2B).

*Gβ-*null cells showed a complete absence of cAMP-evoked GCaMP fluorescence change. It should be noted, however, that this result may not solely reflect a direct requirement for Gβγ signaling in Ca^2+^ channel activation. The absence of a Ca^2+^ response in *Gβ-*null cells may alternatively arise from their inability to undergo proper starvation-induced development. Since *Gβ-*null cells fail to acquire full aggregation-competence, they are likely deficient in cAMP receptors (cARs) and downstream signaling proteins required for cAMP-evoked Ca^2+^ influx. Further studies are warranted to discriminate between these possibilities.

*gca/sgc*-null cells—deficient in both cGMP-producing guanylyl cyclases—displayed a cAMP-induced Ca^2+^ response with peak [Ca^2+^]i occurring at approximately 20–30 sec post-stimulation, a modest delay relative to the ~20-sec peak observed in wild-type cells (Fig. 2B). One-way ANOVA performed at each time point, however, showed no statistically significant difference between the *gca/sgc*-null and wild-type time courses at any individual time point (*P* > 0.05), despite a significant difference detected among all genotypes at 12–16 sec post-stimulation (Fig. 2B). Given this lack of significance in direct pairwise comparison we do not draw a strong conclusion regarding a kinetic role for cGMP in Ca^2+^ channel activation.

Strikingly, *iplA*-null cells showed no detectable change in GCaMP fluorescence in response to cAMP stimulation (Fig. 2A and 2B). Since extracellular Ca^2+^ influx is the obligate source of the signal (Fig. 1), the loss of response in *iplA*-null cells implies that IplA is required for activating plasma membrane Ca^2+^ entry. This observation is consistent with a model in which *IplA-*mediated intracellular Ca^2+^ release depletes ER stores, which in turn signals plasma membrane channel activation through a SOCE-like mechanism [3, 13]. Notably, *plaA*-null cells exhibited cAMP-induced Ca^2+^ dynamics indistinguishable from wild-type cells (Fig. 2A and 2B), confirming that PlaA-mediated phospholipid remodeling is dispensable for this response.

### SERCA Inhibition Elevates Basal [Ca^2+^]i and Abolishes Chemoattractant-Induced Ca^2+^ Responses, Consistent with a Store-Operated Ca^2+^ Entry Mechanism

To investigate the functional role of the sarco/endoplasmic reticulum (sER) Ca^2+^ pool in chemoattractant-evoked responses, we pre-treated wild-type and mutant cells with 50 μM BHQ, a specific and reversible inhibitor of SERCA pumps that prevents Ca^2+^ re-uptake into the sER [15, 16]. In the presence of 50 μM BHQ, neither wild-type cells nor any of the mutant cell lines (*Gβ-*null, *gca/sgc*-null, *iplA*-null, *plaA*-null) showed a detectable increase in GCaMP fluorescence in response to 15 μM cAMP (Fig. 3). This result indicates that functional SERCA-dependent sER Ca^2+^ sequestration is a prerequisite for receptor-stimulated Ca^2+^ influx, suggesting that the ER is the central source of Ca^2+^ mobilization in cAMP-stimulated *Dictyostelium* cells.

Notably, substantially elevated basal GCaMP fluorescence was observed in BHQ-treated cells even before cAMP addition (Fig. 3), indicating that SERCA inhibition results in constitutive [Ca^2+^]i elevation due to uncompensated passive Ca^2+^ leak from the sER. Under chronic SERCA inhibition by BHQ, the sER would be locked in a constitutively depleted state, unable to accumulate the Ca^2+^ load necessary to generate a receptor-stimulated depletion signal for SOCE activation. This interpretation aligns with the SOCE model described in mammalian cells, where agonist-induced IP_3_R-mediated ER store depletion—rather than cytosolic Ca^2+^ itself—is the trigger for STIM1-mediated Orai1 channel activation [3, 17]. Taken together, the BHQ data, combined with the *IplA-*null phenotype in Fig. 2, provide coherent pharmacological and genetic evidence consistent with a SOCE-like mechanism in *Dictyostelium*.

### Differential G-Protein Dependency between cAMP and Folate Ca^2+^ Pathways in Vegetative Cells

To determine whether the signaling requirements identified for cAMP-evoked Ca^2+^ responses in developed cells generalize to the vegetative folate pathway, we examined GCaMP dynamics in wild-type, *Gβ-*null, *gca/sgc*-null, and *iplA*-null vegetative cells stimulated with 100 μM folate (Fig. 4). Wild-type vegetative cells exhibited a transient [Ca^2+^]i elevation with peak fluorescence at approximately 20 seconds post-stimulation (Fig. 4B), comparable to the timing of the cAMP response in developed cells. This is consistent with prior aequorin measurements in vegetative cells [10, 13]. Vegetative *plaA*-null cells were not included in this study, because *plaA*-null cells showed cAMP-induced Ca^2+^ dynamics indistinguishable from wild type (Fig. 2). Confirmation that PlaA is similarly dispensable for the folate response will require direct testing in future work.

The most unexpected result of the present study was observed in *Gβ-*null vegetative cells. Rather than showing abolished or reduced Ca^2+^ responses as reported by Nebl *et al*. (2002) [10] using aequorin, *Gβ-*null cells retained a transient Ca^2+^ response to folate with slightly accelerated kinetics, peaking at approximately 8−12 seconds (Fig. 4B). To confirm this phenotype, we repeated the folate-stimulation experiment on independently prepared *Gβ-*null cells (Fig. 4C, 5 independent experiments) and incorporated these data directly into the pooled dataset shown in the Fig. 4B, supporting the reproducibility of this observation. This result contrasts with the complete abolition of cAMP-induced Ca^2+^ responses in *Gβ-*null developed cells (Fig. 2). However, because the *Gβ-*null cells used for the cAMP experiments were developmentally compromised, we cannot fully exclude the possibility that the asymmetry between the cAMP and folate results partly reflects differences in developmental context rather than a direct, opposing regulatory role of Gβ itself. We also note that our result diverges from the aequorin-based measurements of Nebl *et al*. (2002) [10]. This discrepancy may reflect differences between the two reporter systems, including their relative Ca^2+^ affinities and kinetics, differences in expression level and subcellular targeting, and the biochemical reconstitution requirements of aequorin (which depends on exogenous coelenterazine). *gca/sgc*-null vegetative cells displayed a folate-induced Ca^2+^ response with higher amplitude compared to wild-type cells (Fig. 4B), suggesting that cGMP plays different regulatory roles in the two chemoattractant pathways. Critically, *iplA*-null vegetative cells showed no detectable [Ca^2+^]i response to folate stimulation (Fig. 4B), demonstrating that IplA is absolutely required for chemoattractant-induced Ca^2+^ signaling regardless of chemoattractant identity or developmental stage.

### Calmodulin Activity Is Not Required for Ca^2+^ Influx

Calmodulin (CaM) is the primary intracellular Ca^2+^ sensor that transduces [Ca^2+^]i elevations into downstream responses, including regulation of contractile vacuole (CV) function and myosin II-dependent cytoskeletal reorganization [2, 3, 18]. To assess whether CaM-mediated processes are required for the Ca^2+^ influx response itself, we pre-treated cells with W7 (N-(6-aminohexyl)-5-chloro-1-naphthalenesulfonamide), a well-characterized calmodulin antagonist, and then stimulated with cAMP (Fig. S1). Control (Con) cells were incubated in DB buffer without any drug or DMSO addition for the same 1 h period. In the presence of W7, all cells including wild-type cells and mutant cells showed similar calcium induction dynamics in response to cAMP stimulation compared to those in the absence of W7 (Fig. S1).

These results indicate that CaM activity is not required for the upstream signaling events that gate Ca^2+^ influx and that contractile vacuoles do not make an essential contribution to the observed cAMP-induced [Ca^2+^]i transient. This conclusion is consistent with the findings of Wilczynska *et al*. (2005) [13], who demonstrated that ER Ca^2+^ stores—rather than CV or other acidic compartments—are the primary source of chemoattractant-induced cytosolic Ca^2+^ elevations. The slight reduction in Ca^2+^ response amplitude observed in W7-treated wild-type cells suggests that CaM-dependent processes may contribute modestly to the secondary amplification of the Ca^2+^ signal, consistent with the model proposed by Kim *et al*. (2025) [7].

## Discussion

In this study, we employed the genetically encoded Ca^2+^ biosensors GCaMP3 to provide a comprehensive real-time characterization of chemoattractant-induced [Ca^2+^]i dynamics in *Dictyostelium discoideum*. Through systematic analysis of defined buffer conditions, pharmacological perturbations, and a panel of signaling mutants, we have established the molecular architecture of Ca^2+^ mobilization in both cAMP-stimulated developed cells and folate-stimulated vegetative cells. Our data confirm several previously established principles, reveal important new regulatory features, and identify key areas for future investigation.

The complete abrogation of GCaMP signals under Ca^2+^-free conditions provides strong evidence that plasma membrane Ca^2+^ influx—not release from intracellular stores alone—is the primary driver of chemoattractant-evoked [Ca^2+^]i transients. This conclusion advances prior radioisotope-based evidence by providing single-cell temporal resolution [4, 19, 21]. A numerically higher Ca^2+^ response was observed in Mg^2+^-free buffer, consistent with the trend reported by Hong *et al*. (2026) [20]; however, in our pairwise comparison this trend did not reach statistical significance (*P* > 0.05), and we therefore regard a possible modulatory role of extracellular Mg^2+^ as suggestive rather than established. Further comparative studies will be required to determine whether Mg^2+^ significantly modulates this Ca^2+^ influx pathway.

The complete loss of Ca^2+^ responses in *iplA*-null cells under both cAMP and folate stimulation—despite the obligatory requirement for extracellular Ca^2+^ influx—is one of the most mechanistically informative findings of this study. IplA, a distant IP_3_R homolog, has previously been implicated in *Dictyostelium* chemotaxis and electrotaxis [4, 22, 23] but has not been established as an essential mediator of chemotaxis Ca^2+^ responses across developmental stages. Our data now demonstrate this conserved essential role and suggest that *IplA-*mediated ER Ca^2+^ release is required not only for intracellular Ca^2+^ release but also for activating plasma membrane Ca^2+^ influx, consistent with a SOCE-like coupling mechanism.

The BHQ experiments provide independent pharmacological support for this model. SERCA inhibition by BHQ not only depleted the ER Ca^2+^ store (evidenced by elevated basal [Ca^2+^]i) but also completely abolished receptor-stimulated Ca^2+^ influx (Fig. 3). Under BHQ treatment, the SERCA-inhibited ER cannot accumulate sufficient Ca^2+^ to generate a meaningful depletion signal upon stimulation. This interpretation is further supported by the findings of Wilczynska *et al*. (2005) [13]., who demonstrated that ER Ca^2+^-binding proteins calreticulin and calnexin regulate the amplitude and kinetics of Ca^2+^ responses by modulating the ER Ca^2+^ gradient. A mechanistic parallel is provided by Yamada *et al*. (2025) [24], who showed in zebrafish epithelia that IP_3_R-mediated CICR amplifies mechanosensitive Ca^2+^ influx. Future work should aim to identify functional STIM and Orai homologs in *Dictyostelium* to directly test the SOCE hypothesis.

One of the most notable observations of this study is the difference in Gβ dependence between the cAMP and folate Ca^2+^ pathways. While *Gβ-*null developed cells show no cAMP-induced Ca^2+^ response, it should be noted that this absence may not solely reflect a direct requirement for Gβγ in Ca^2+^ channel activation. Because *Gβ-*null cells fail to properly complete starvation-induced development, they are likely deficient in cAMP receptors (cARs) and associated downstream signaling proteins, which could independently account for the lack of a Ca^2+^ response. In contrast, *Gβ-*null vegetative cells, which do not require starvation-induced development to respond to folate, exhibited a faster folate-induced Ca^2+^ response peaking at approximately 8-12 sec. This raises the possibility that Gβ normally dampens the folate-pathway Ca^2+^ response and that the folate receptor engages a pathway that does not require Gβ in vegetative cells. This apparent divergence from earlier aequorin-based reports (Nebl *et al*., 2002 [10]) may additionally reflect differences in reporter sensitivity, kinetics, and reconstitution requirements between aequorin and GCaMP3, as discussed above.

The higher response amplitude in *gca/sgc*-null cells upon folate stimulation suggests pathway-specific regulatory logic in which cGMP limits Ca^2+^ influx amplitude in the vegetative folate context. Our W7 data indicate that CaM-dependent processes, including Ca^2+^ release from contractile vacuoles (CV), are not essential for the cAMP-induced Ca^2+^ transient, supporting the primacy of ER-derived Ca^2+^ in chemoattractant signaling and consistent with the genetic evidence of Wilczynska *et al*. (2005) [13].

Several methodological considerations should also be noted when interpreting the kinetic comparisons reported here. First, chemoattractant stimulation was induced by manual pipetting of a 1/10 volume of chemoattractant into an imaging dish. Based on the time required for visible mixing under these conditions, the delivery/mixing latency is estimated to be within approximately 1 sec. While this is shorter than most reported maximum response times (~20 sec), it holds relatively greater significance for the fastest observed response (*Gβ-*null cells upon folate, ~8 seconds). Future studies using microfluidic perfusion or automated high-speed mixing devices would allow for a more accurate analysis of these particularly fast response rate characteristics. Second, GCaMP3 is a single-wavelength, non-ratiometric indicator, and because both cAMP and folate stimulation trigger rapid morphological and pseudopod changes within the imaging window, changes in cell shape, volume, or focal plane could in principle contribute to apparent fluorescence changes independent of true [Ca^2+^]i change. The absence of any fluorescence change in empty GFP-expressing cells (Fig. 1A) argues against a major contribution of shape/volume artifacts to the GCaMP signal, but we cannot fully exclude a modest contribution to the smaller-amplitude or faster-kinetics comparisons, and incorporating such a control is an important goal for future work. Third, the W7 and BHQ stock solutions were prepared in DMSO and diluted into the imaging buffer to a final concentration of 50 μM drug. The corresponding control (“Con”) condition in this study consisted of cells incubated in drug-free DB buffer rather than a matched DMSO-only vehicle. Because the final DMSO carryover at this dilution is very low (estimated <0.2%), we do not believe this affected our conclusions. However, we note that a dedicated DMSO-only vehicle control was not included, and this would be incorporated in future experiments using these reagents.

In conclusion, this study provides a comprehensive molecular dissection of chemoattractant-induced Ca^2+^ signaling in *Dictyostelium* using GCaMP-based live-cell imaging. Our key conclusions are: (1) extracellular Ca^2+^ influx is obligatory for both cAMP and folate Ca^2+^ responses, with a non-significant trend toward enhancement in the absence of extracellular Mg^2+^ that requires further validation; (2) IplA is universally required across developmental stages and chemoattractant systems; (3) SERCA-dependent ER Ca^2+^ loading is required for receptor-stimulated Ca^2+^ influx, providing pharmacological and genetic evidence consistent with a SOCE-like mechanism; (4) vegetative *Gβ-*null cells exhibit a folate-induced Ca^2+^ response, raising the possibility of a *Gβ-*independent Ca^2+^ entry pathway; and (5) pharmacological inhibition of CaM did not block the cAMP-induced Ca^2+^ response, indicating that CaM activity is not required for the generation of this Ca^2+^ influx , with ER stores—rather than contractile vacuoles-providing the primary Ca^2+^ source; whether CaM subsequently functions as a downstream Ca^2+^ effector was not directly examined in this study and remains to be established.

## Supplemental Materials

Supplementary data for this paper are available on-line only at http://jmb.or.kr.



## Figures and Tables

**Fig. 1:**
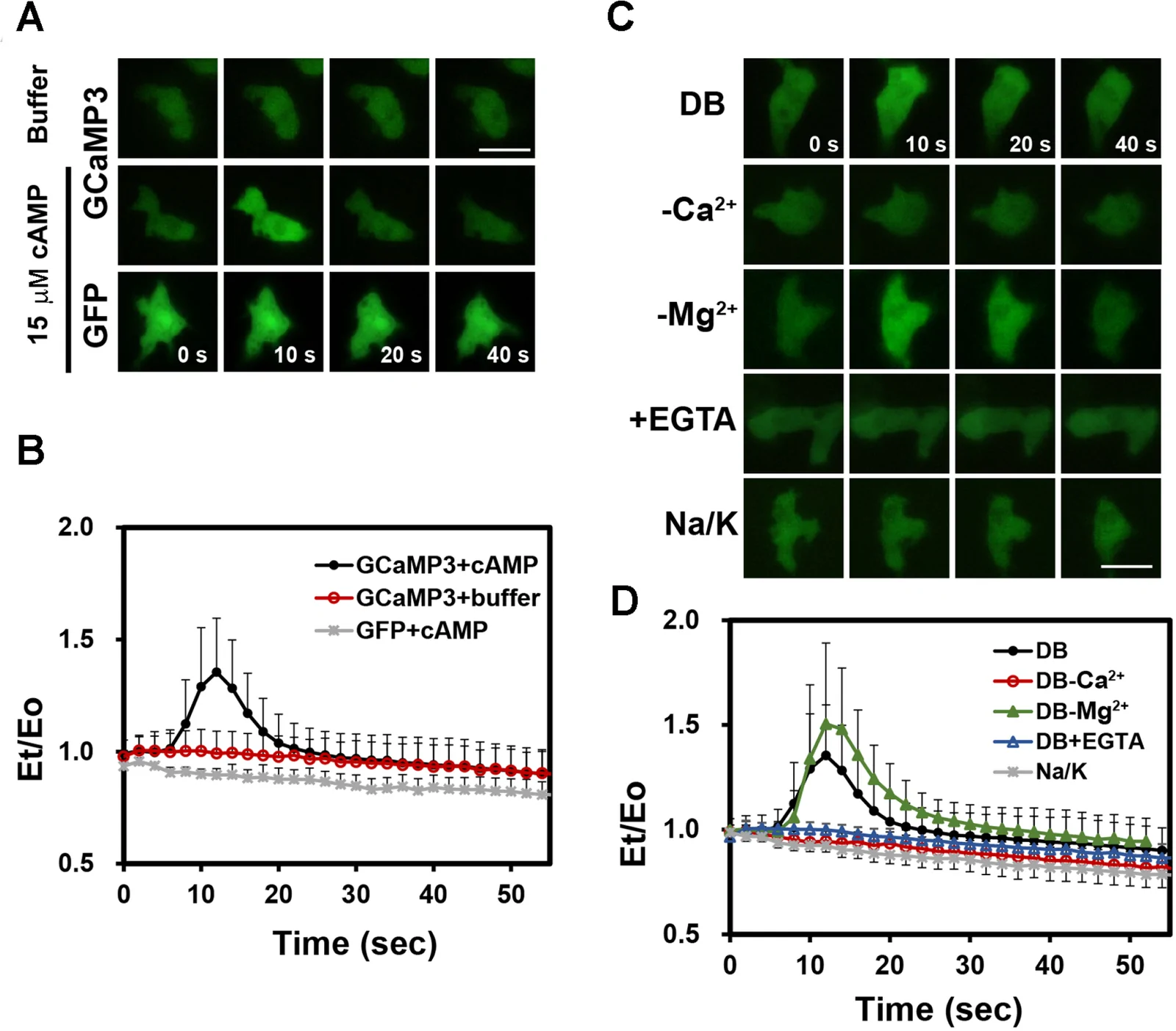
cAMP-induced intracellular [Ca^2+^]i response in wild-type cells. (**A**) [Ca^2+^]i response of wild-type cells upon cAMP stimulation. Wild-type cells expressing pDXA-GCaMP3 were uniformly stimulated with 15 μM cAMP in DB buffer (labeled “GCaMP3 + cAMP”, second row). Cells expressing empty GFP were used as a control (labeled “GFP + cAMP”, third row), and DB buffer without cAMP stimulation was used as a negative stimulation control (labeled “GCaMP3 + buffer”, first row). Fluorescence images were taken after stimulation for 1 min with 2-sec intervals, and representative images at the indicated time points are shown. Scale bar = 5 μm. (**B**) Dynamics of calcium levels in wild-type cells in response to cAMP stimulation. Fluorescence intensities (Et/Eo, see Materials and Methods) were measured and quantified from time-lapse recordings using the ROI manager in ImageJ. Each condition is plotted in a distinct color as indicated in the legend. The graphs represent the mean values of multiple cells per condition, pooled from 3 independent experiments (GCaMP3 + cAMP, N = 160; GFP + cAMP”, N = 89; GCaMP3 + buffer, N = 50), N = 3 independent experiments; see Materials and Methods, Statistical analysis, for the experiment-level comparison approach). Error bars represent SD. (**C**) [Ca^2+^]i response of wild-type cells upon cAMP stimulation in various conditions. Cells expressing GCaMP3 were uniformly stimulated with cAMP in DB buffer (5 mM Na_2_HPO_4_, 5 mM KH_2_PO_4_, 2 mM MgCl_2_, and 1 mM CaCl_2_), DB buffer without CaCl_2_ (DB-Ca^2+^), DB buffer without MgCl_2_ (DB-Mg^2+^), DB buffer with EGTA (DB+EGTA), or Na/K phosphate buffer (Na/K). (**D**) Dynamics of calcium levels in response to cAMP stimulation in various conditions. Fluorescence intensities were measured and quantified as described above. The graphs represent the mean values of multiple cells per condition, pooled from 3 independent experiments (DB, N = 160; DB-Ca^2+^, N = 82; DB-Mg^2+^, N = 118; DB+EGTA, N=60; Na/K, N = 60). For panel (**B**), one-way ANOVA performed at each 2-sec time point revealed statistically significant differences among conditions at 12–14 sec post-stimulation (*P* < 0.05). For panel (**D**), one-way ANOVA at each 2-sec time point showed statistically significant differences among conditions at 10–14 sec post-stimulation (*P* < 0.05); pairwise comparison between the DB and DB-Mg^2+^ conditions did not reach statistical significance at any time point (*P* > 0.05).

**Fig. 2:**
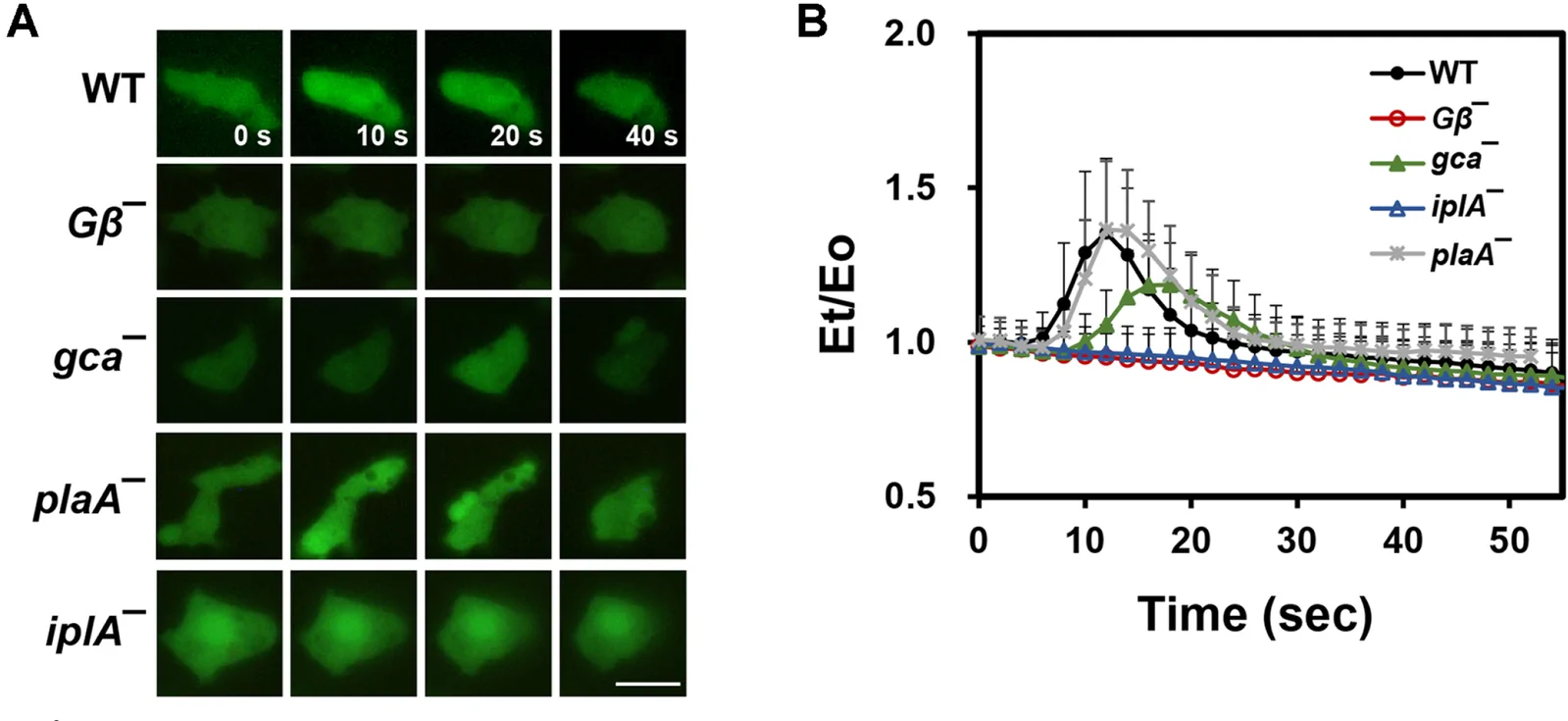
cAMP-induced [Ca^2+^]i response in mutant cells. (**A**) [Ca^2+^]i response of mutant cells in response to cAMP stimulation. Wild-type cells and mutant cells lacking Gβ, GCA/sGC, IplA or PlaA, all expressing pDXA-GCaMP3, were uniformly stimulated with 15 μM cAMP. Fluorescence images were taken after stimulating for 1 min with 2-sec intervals, and representative images at the indicated time points were presented. Scale bar = 5 μm. (**B**) Dynamics of calcium levels in mutant cells, each plotted in a distinct color as indicated in the legend, in response to cAMP stimulation. Fluorescence intensities in mutant cells were measured and quantified from time-lapse recordings using the ROI manager in ImageJ. Graphs represent the mean values of multiple cells per genotype (Wild-type, N = 160; *Gβ*^−^, N = 105; *gca*^−^, N = 138; *iplA*^−^, N = 68; *plaA*^−^, N = 119), pooled from N = 3 independent experiments per genotype. Error bars indicate SD. Peak time and peak amplitude were compared across genotypes by one-way ANOVA with Tukey's post hoc test on experiment-level means (see Materials and Methods). One-way ANOVA performed at each 2-sec time point on the fluorescence intensity time courses revealed statistically significant differences among genotypes at 12–16 sec post-stimulation (*P* < 0.05). Tukey post hoc pairwise comparisons on experiment-level means showed that neither the *gca/sgc*-null nor the *plaA*-null time course differed significantly from wild-type at any time point (both *P* > 0.05), whereas *Gβ-*null and *iplA*-null cells, which showed no detectable response, differed significantly from wild-type across the majority of the time course (*P* < 0.05).

**Fig. 3:**
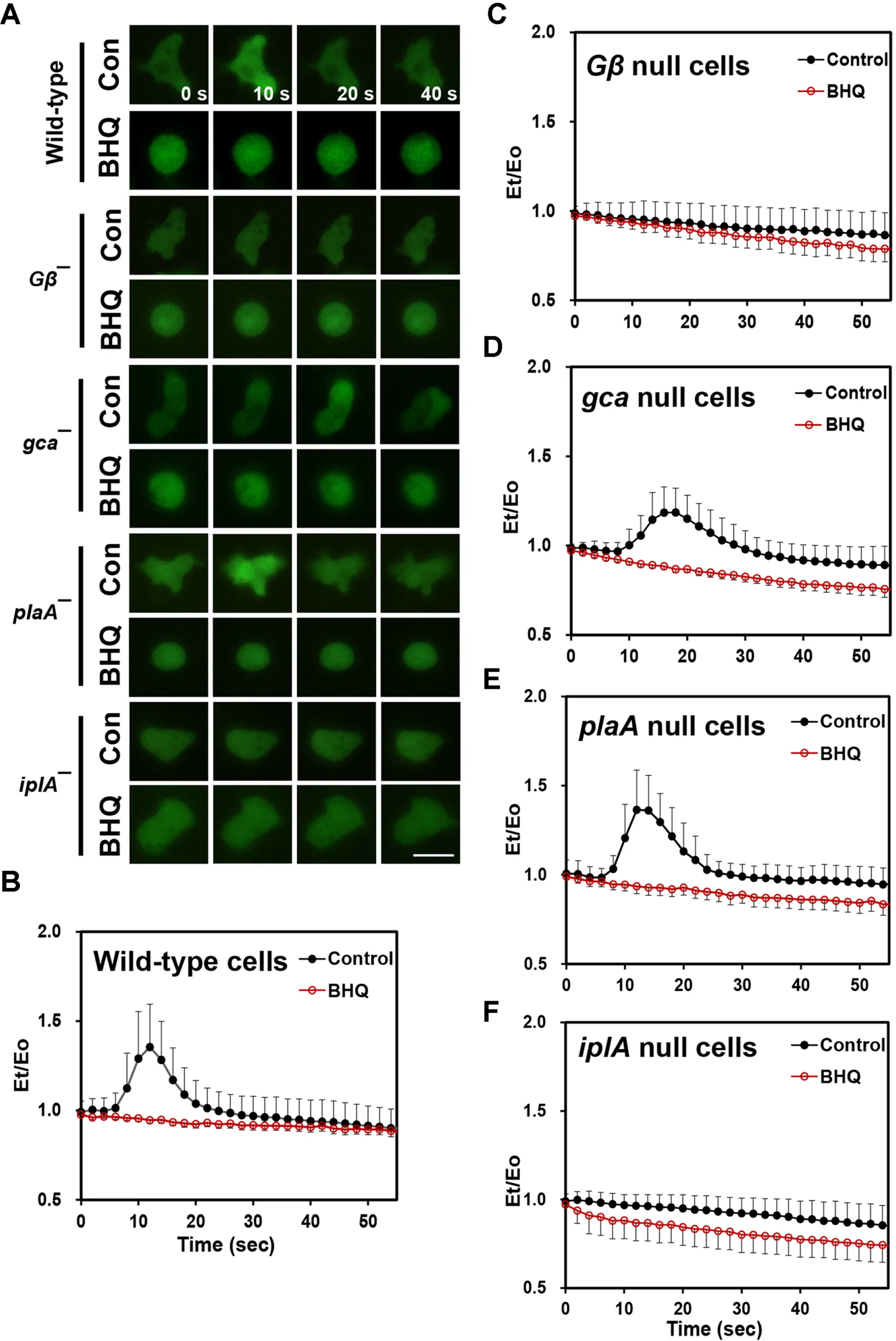
cAMP-induced [Ca^2+^]i responses in the presence of BHQ inhibitors. (**A**) [Ca^2+^]i response of cells in the presence of BHQ inhibitors. Wild-type and mutant cells expressing GCaMP3 were incubated with or without 50 μM BHQ for 1 h and then stimulated with 15 μM cAMP. Fluorescence images were taken after stimulating the cells, and representative images at the indicated time points are presented. Scale bar = 5 μm. Dynamics of calcium levels in wild-type cells (**B**), *Gβ-*null cells (**C**), *gca/sgc*-null cells (**D**), *plaA*-null cells (**E**), and *iplA*-null cells (**F**) in the presence (distinct color) or absence (distinct color, as indicated in each panel legend) of BHQ. Fluorescence intensities of the cells in the presence of BHQ were measured as described in Fig. 2 and compared with those of the cells in the absence of BHQ as a control. Graphs represent the mean values of multiple cells per condition, pooled from N = 3 independent experiments (Wild-type, Control N = 160, BHQ N = 54; *Gβ*^−^, Control N = 105, BHQ N = 60; *gca*^−^, Control N = 138, BHQ N = 66; *iplA*^−^, Control N = 68, BHQ N = 51; *plaA*^−^, Control N = 119, BHQ N = 58); BHQ versus no-BHQ comparisons within each genotype were performed by two-tailed Student's t-test on experiment-level means. Error bars indicate SD. This comparison revealed statistically significant differences between BHQ-treated and untreated cells in wild-type, *gca/sgc*-null, and *plaA*-null cells (*P* < 0.05), whereas no statistically significant difference was detected in *Gβ-*null or *iplA*-null cells (*P* > 0.05).

**Fig. 4:**
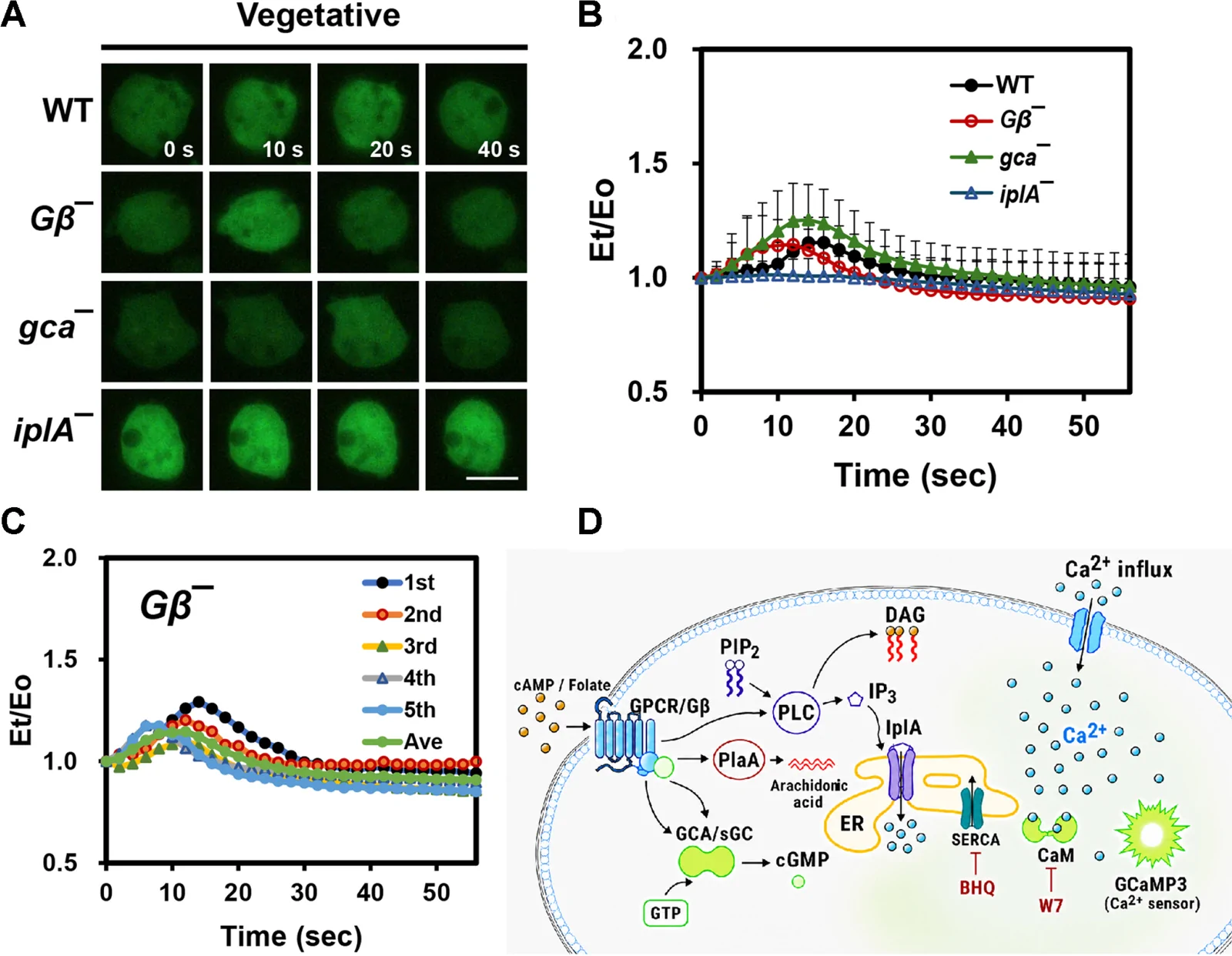
Folate-induced [Ca^2+^]i response in vegetative cells and proposed model of chemoattractant-induced Ca^2+^ signaling. (**A**) [Ca^2+^]i response of vegetative cells expressing GCaMP3 in response to folate stimulation. Wild-type cells and mutant cells lacking Gβ, GCA/sGC, or IplA were uniformly stimulated with 100 μM folate. Fluorescence images were taken at 2-second intervals for 1 min after stimulation, and representative images at the indicated time points were presented. Scale bar = 5 μm. (**B**) Dynamics of calcium levels in mutant cells, each plotted in a distinct color as indicated in the legend, in response to folate stimulation. Fluorescence intensities in mutant cells were measured and quantified from time-lapse recordings using the ROI manager in ImageJ. Graphs represent the mean values of multiple cells per genotype, pooled from N = 3 independent experiments per genotype (Wild-type, N = 56; *Gβ*^−^, N = 220; *gca*^−^, N = 64; *iplA*^−^, N = 89). One-way ANOVA test performed at each 2-sec time point demonstrated statistically significant differences between wild-type and *Gβ-*null cells at 18–60 sec post-stimulation, and between wild-type and *gca/sgc*-null cells at 10–20 sec post-stimulation (*P* < 0.05). Error bars indicate SD. (**C**) Folate-induced [Ca^2+^]i response of vegetative *Gβ-*null cells across independently prepared biological replicates. To further verify the reproducibility of the accelerated *Gβ-*null Ca^2+^ transient shown in (**B**), vegetative *Gβ-*null cells expressing GCaMP3 were stimulated with 100 μM folate in five independently prepared experiments (Experiment 1, N = 119; Experiment 2, N = 38; Experiment 3, N = 17; Experiment 4, N = 30; Experiment 5, N = 16 cells; total pooled N = 220 cells from N = 5 independent experiments). Fluorescence intensities were measured and quantified as described above. Individual traces from each replicate are shown, demonstrating consistent response kinetics and amplitude across all five independent experiments. (**D**) Proposed model of chemoattractant-induced Ca^2+^ signaling in *Dictyostelium*. Upon chemoattractant binding, the GPCR/Gβ complex activates phospholipase C (PLC), which hydrolyzes PIP2 into DAG and IP3; IP3 acts on the ER-localized IplA channel to release Ca^2+^ from the ER lumen into the cytosol. GPCR/Gβ signaling also activates PlaA, generating arachidonic acid, and GCA/sGC, generating cGMP, both of which modulate the kinetics and magnitude of the cytosolic Ca^2+^ response. ER Ca^2+^ release via IplA is coupled to Ca^2+^ influx across the plasma membrane through a store-operated-like mechanism that requires SERCA-dependent ER Ca^2+^ reuptake (sensitive to BHQ). The resulting rise in cytosolic Ca^2+^ is detected by the genetically encoded sensor GCaMP3 and can also engage calmodulin (CaM, sensitive to W7) as a downstream effector.
