# Supplementary material for: Chemoattractant-Induced Intracellular Calcium Responses Using a Calcium Biosensor GCaMP3 in Dictyostelium discoideum
Source: J Microbiol Biotechnol. 2026 Oct 21;36:e2607011. doi: 10.4014/jmb.2607.07011 (PMC13696953; doi:10.4014/jmb.2607.07011)
Supplement: Supplementary file 1 [file jmb-36-e2607011-supple.pdf]

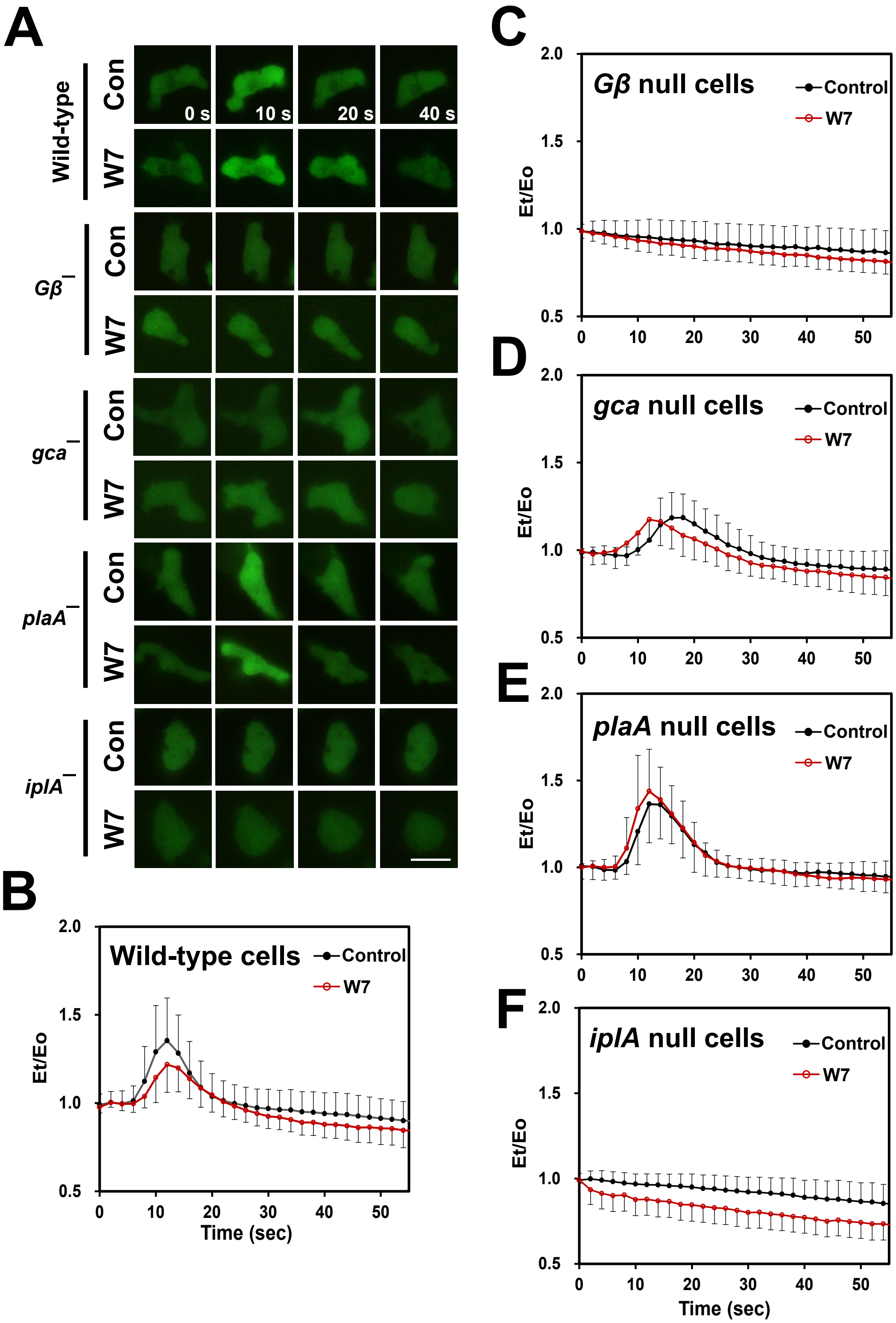

Supp.Figure 1. Kim et al
